# Prediction of occult lymph node metastasis using volume-based PET parameters in small-sized peripheral non-small cell lung cancer

**DOI:** 10.1186/s40644-015-0058-9

**Published:** 2015-12-22

**Authors:** Seong Yong Park, Joon-Kee Yoon, Kwang Joo Park, Su Jin Lee

**Affiliations:** Department of Thoracic and Cardiovascular Surgery, Ajou University School of Medicine, Suwon, Korea; Department of Nuclear Medicine and Molecular Imaging, Ajou University School of Medicine, 164, Worldcup-ro, Yeongtong-gu, Suwon 443-380 Korea; Division of Pulmonary and Critical Care Medicine, Department of Medicine, Ajou University School of Medicine, Suwon, Korea

**Keywords:** Non-small cell lung cancer, Fluorine 18-fluorodeoxyglucose (FDG), Positron emission tomography/computed tomography (PET/CT), Lymph node metastasis, Metabolic tumor volume

## Abstract

**Background:**

Patients with small-sized peripheral non-small cell lung cancer (NSCLC), but without lymph node metastasis, may be optimal candidates for sublobar resection. We aim to identify the predictors of occult lymph node metastasis (OLNM) using F-18 fluorodeoxyglucose positron emission tomography/computed tomography (PET/CT) in clinically node-negative, small-sized NSCLC.

**Methods:**

One hundred thirty nine patients with small-sized NSCLC (of less than 3 cm in diameter) who underwent surgical resection with mediastinal lymph node dissection were evaluated. The maximum standardized uptake value (SUVmax), metabolic total volume (MTV) and total lesion glycolysis (TLG) of the primary tumor were measured on pretreatment PET/CT. These metabolic parameters and pathological variables were analyzed for OLNM.

**Results:**

The mean tumor size was 2.11 ± 0.63 cm, and the mean number of dissected lymph nodes was 19.74 ± 12.86. Adenocarcinoma occurred in 106 patients (76.3 %). Twenty-four patients (17.2 %) had lymph node metastasis. The mean SUVmax, MTV and TLG were 4.61 ± 3.99 (0.5 ~ 17.8), 4.18 ± 6.39 (0 ~ 34.6) and 16.13 ± 28.86 (0 ~ 164.2), respectively. On receiver operating characteristic (ROC) curve analysis, the areas under the curve (AUC) for SUVmax, MTV and TLG for node metastasis were 0.753, 0.783 and 0.775, respectively. On multivariate analysis, SUVmax (Odds ratio [OR] = 1.120, p = 0.044) and MTV (OR = 1.117, p = 0.007) were found to be risk factors for OLNM. The concordance index of MTV was 0.763, which was slightly higher than that of SUVmax.

**Conclusion:**

SUVmax and volume-based parameters are significant risk factors for OLNM in small peripheral NSCLC. MTV showed a better predictive performance than that of the other PET parameters; therefore, MTV may be a possible indicator for sublobar resection in clinically node-negative small-sized NSCLC.

## Background

The early detection of small-sized non-small cell lung cancer (NSCLC) has increased due to recent advances in radiographic technologies, such as high-resolution computed tomography (CT) and the widespread use of low-dose helical CT for screening [1–3]. Even though anatomic resection, such as lobectomy and mediastinal lymph node dissection, is the surgical treatment of choice for NSCLC, sublobar resections such as segmentectomy and wedge resection have also been performed to preserve lung function. Several studies have reported that the survival duration is similar between patients with small peripheral NSCLC treated with segmentectomy versus lobectomy [4–7]. In determining the indications for sublobar resection, prediction of the pathological node-negative (pN0) status is an important factor; if nodal metastasis is present, lobectomy and mediastinal lymph node dissection rather than sublobar resection are mandatory. According to previous studies, tumor size, SUVmax on PET/CT and tumor type, such as adenocarcinoma, have been suggested as risk factors for node metastasis in early NSCLC [8].

^18^F-fluorodeoxyglucose positron emission tomography (FDG PET) is a valuable imaging modality for staging, management and predicting the prognosis of NSCLC. FDG PET parameters, standardized uptake value (SUV), metabolic tumor volume (MTV) and total lesion glycolysis (TLG), can provide useful data on tumor metabolism. Whereas the maximum SUV (SUVmax) is the value of a single voxel with the highest radiotracer concentration within the tumor, volume-based parameters such as MTV and TLG indicate tumor burden. There has been a growing interest in the clinical significance of volume-based parameters for NSCLC. Studies have shown that volume parameters are a better index of patient prognosis than is SUVmax in advanced NSCLC [9–11]. In addition, recent studies have shown that volume parameters are also a significant prognostic factor in early stage NSCLC [12–14]. The most recent study reported the prognostic role of TLG in stage IA NSCLC [14]. Based on these previous studies regarding PET volume parameters, we hypothesized that the volume parameter of FDG PET can also predict node metastasis in early NSCLC. Even though several studies have reported that the SUVmax of a primary tumor is a significant predictor of node metastasis in clinical stage IA lung cancer [8, 15], there have been few studies using volume-based parameters for detecting occult node metastasis in early-stage lung cancer.

In this study, therefore, we investigated the risk factors for node metastasis in clinically node-negative, small peripheral NSCLC. The predictive roles of PET parameters, SUVmax and volume-based parameters were also evaluated and were compared using statistical methods.

## Methods

### Patients

We retrospectively reviewed the medical records of 440 consecutive NSCLC patients who underwent both pretreatment FDG PET/CT and surgery between January 2010 and June 2015 at the Ajou Medical Center. All patients underwent chest CT, bronchoscopy, FDG PET/CT and brain magnetic resonance imaging for staging work-up. The primary tumor size was measured on chest CT images. The inclusion criteria were a small NSCLC of less than 3 cm, located in the outer one-third of the lung parenchyma, with no enlargement of the interlobar, hilar or mediastinal lymph nodes on chest CT and no significant FDG uptake in the lymph nodes except for the primary lesion. We subsequently evaluated 139 patients with small-sized and peripheral NSCLC. The ethics committee of our institution approved this retrospective study (IRB No. MED-MDB-13-391), and requirement for informed consent was waived.

### FDG PET-CT protocol

FDG PET/CT was performed using two types of PET/CT scanners: the Discovery ST and the Discovery STE (GE healthcare, Milwaukee, WI, USA). All patients fasted for at least 6 hours before scanning; serum glucose levels at the time of FDG injection were < 150 mg/dl. Unenhanced CT was performed 60 min after a 5 MBq/kg FDG injection using 8- or 16-slice helical CT (120 keV, 30–100 mA in AutomA mode; section width = 3.75 mm). Emission PET data was acquired from the thigh to the head for 2.5 min per frame in the three-dimensional mode. Attenuation-corrected PET images using CT data were reconstructed by an ordered-subsets expectation maximization algorithm (20 subsets, 2 iterations).

### Measurement of PET parameters

The measurement of PET parameters was performed using a dedicated workstation (GE Advantage Workstation 4.4; GE Healthcare). We measured the various PET parameters of the primary lung lesion. Volume viewer software, which represents the volume of interest (VOI) automatically delimited by an isocontour threshold method based on the SUV, was used to calculate metabolic parameters including SUVmax, average SUV (SUVavg), MTV and TLG. The SUVmax was defined as the voxel with the highest count within the VOI. Fixed SUV values (1.5, 2.0, 2.5 and 3.0) were used to define VOI boundaries. MTVs were automatically calculated by summing the total volumes of voxels with threshold SUVs of 1.5, 2.0, 2.5 and 3.0 in the VOI, respectively. TLG was calculated by multiplying the SUVavg by the MTV.

### Statistical analysis

Statistical analysis was performed using the open source statistical software R (http://www.R-project.org). Clinical and pathological parameters were described as means ± standard deviations of the mean for continuous variables and as frequencies (%) for categorical variables. For the purpose of statistical analysis, clinical variables were grouped into two categories with the exception of tumor size, PET parameters and the number of dissected lymph nodes. Receiver operating characteristic (ROC) analysis was performed to calculate the area under the ROC curve (AUC) for each PET parameter. Univariate and multivariate logistic regression models were used to identify the risk factors for occult nodal metastasis. Odds ratios (ORs) with 95 % confidence intervals (CIs) were determined in the multivariate logistic regression model. Preoperative predictors with a *p* < 0.25 in the univariate analysis were entered into a multivariate logistic regression model. The “PredictABEL” packages of R software for performance assessment, calculation of concordance index (C-index) and comparison of several multivariate logistic regression models were used for analyses [16]. All *p*-values were two-sided, and *p* < 0.05 was considered significant.

## Results

### Patient demographics and preoperative PET analysis

**Fig. 1:**
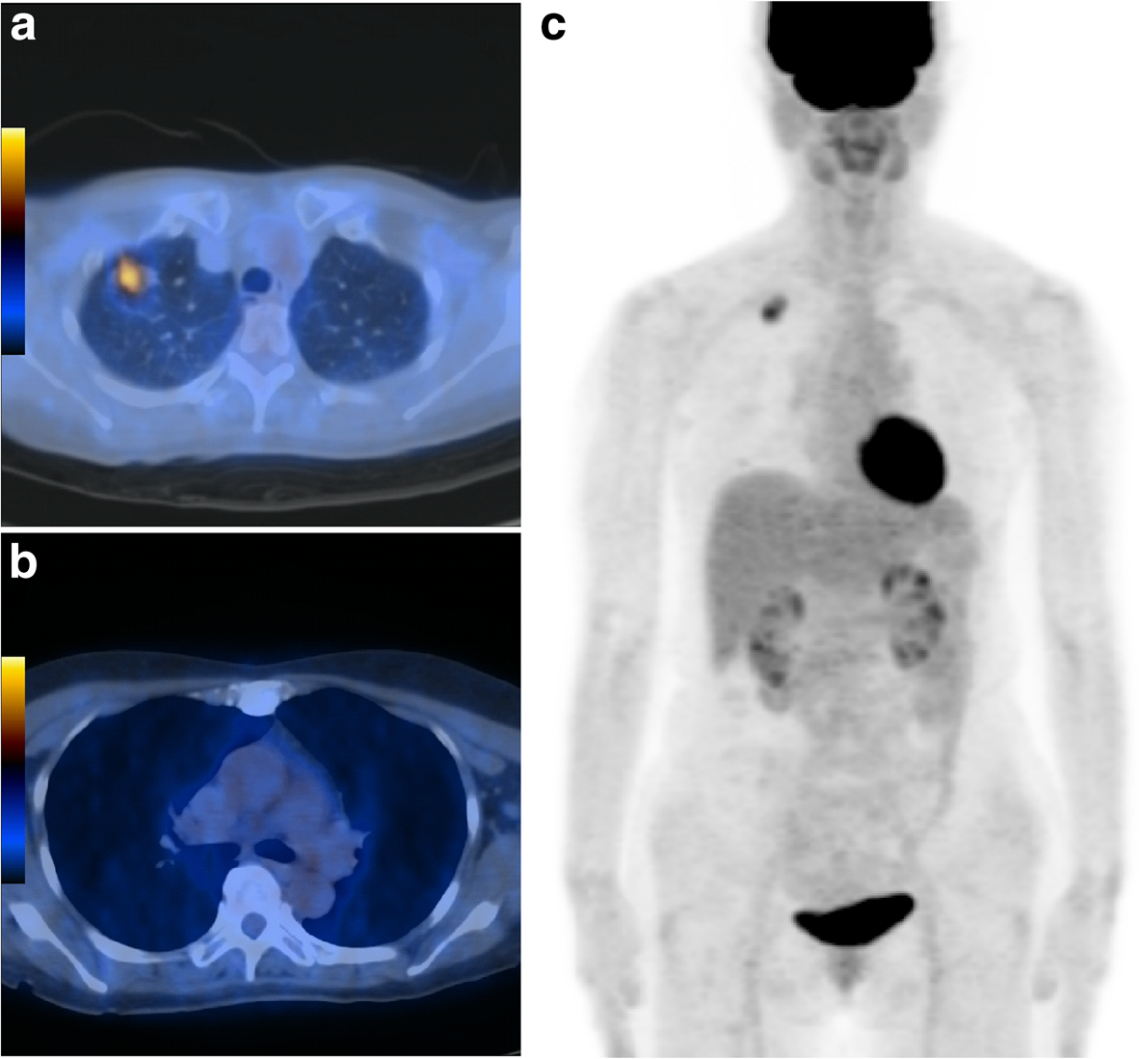
Images of a 60-year-old female patient with adenocarcinoma. The primary tumor size measured on chest CT was 2.4 cm. **a** Transaxial PET/CT fusion image shows a hypermetabolic pulmonary nodule in the right upper lobe. The SUVmax, MTV and TLG of the primary tumor were 4.3, 3.5 cm^3^, and 9.9, respectively. **b** and **c** Transaxial PET/CT fusion image and maximum-intensity-projection image showing no significant FDG uptake suggestive of metastasis; however, right hilar lymph node metastasis was confirmed after surgery

The demographic data of the patients included in this study are listed in Table 1. Of the 139 patients who were evaluated retrospectively, 80 (57.6 %) were male and 59 (42.4 %) were female, with a mean age of 62.82 ± 10.04 years. The mean whole tumor size was 2.11 ± 0.63 cm. The majority of patients underwent anatomic resection (lobectomy or above). The mean number of dissected lymph nodes was 19.74 ± 12.86. The number of adenocarcinomas was 106 (76.3 %). Twenty-four patients (17.2 %) had lymph node metastasis; 12 (8.6 %) were N1 and 12 (8.6 %) were N2. The general characteristics between patients with pathologic N0 and patients with pathologic N1, 2 were not significantly different (Table 1). Figure 1 shows representative PET/CT images of occult lymph node metastasis in small-sized peripheral NSCLC.

**Table 1:** General characteristics of 139 patients

Variables	All patients (*n* = 139)	Pathologic N0 (*n* = 115)	Pathologic N1 and N2 (*n* = 24)	*p* ^*a*^
Age (years)	62.82 ± 10.04 (35 ~ 84)	62.90 ± 10.0	62.46 ± 10.44	0.847
Gender (Male/Female)	80 (57.6 %)/59 (42.4 %)	63 (54.8 %)/52 (45.2 %)	17 (70.8 %)/7 (29.2 %)	0.177
Dissected lymph nodes	19.74 ± 12.86	19.12 ± 12.98	22.67 ± 12.07	0.222
Tumor size (cm)	2.11 ± 0.63	2.05 ± 0.64	2.38 ± 0.57	0.023
Location				0.775
RUL/RML/RLL	54 (38.9 %)/19 (13.7 %)/28 (20.1 %)	46 (40.0 %)/17 (14.8 %)/22 (19.1 %)	8 (33.3 %)/2 (8.3 %)/6 (25.0 %)	
LUL/LLL	26 (18.7 %)/12 (8.6 %)	20 (17.4 %)/10 (8.7 %)	6 (25.0 %)/2 (8.3 %)	
Type of operation				0.161
Wedge resection/Segmentectomy	10 (7.2 %)/2 (1.4 %)	10 (8.7 %)/1 (0.9 %)	0/1 (4.2 %)	
Lobectomy or above	129 (91.4 %)	104 (90.4 %)	23 (95.8 %)	
Pathology				0.290
Adenocarcinoma	106 (76.3 %)	90 (78.3 %)	16 (66.7 %)	
Non-adenocarcinoma	33 (23.7 %)	25 (21.7 %)	8 (33.3 %)	
Differentiation				0.317
Well differentiated	32 (23.1 %)	29 (25.2 %)	3 (12.5 %)	
Moderate differentiated	56 (43.3 %)	45 (39.2 %)	11 (45.8 %)	
Poorly differentiated	17 (12.2 %)	12 (10.4 %)	5 (20.8 %)	
Not defined	34 (24.4 %)	29 (25.2 %)	5 (20.8 %)	
pT stage				0.627
T1a/T1b	43 (31.0 %)/33 (23.7 %)	37 (32.2 %)/28 (24.3 %)	6 (25.0 %)/5 (20.8 %)	
T2a	63 (45.3 %)	50 (43.5 %)	13 (54.2 %)	

^a^comparison between pathologic N0 and pathologic N1, 2

ROC curve analysis was performed to define the optimal cut-off value for measuring the volume-based parameters. Because the AUCs of MTV and TLG measured using a fixed SUV value of 2.0 were higher than the AUCs of MTV and TLG measured using a fixed SUV value of 1.5, 2.5 and 3.0, MTV and TLG measured using SUV 2.0 was adopted (Table 2). The mean SUVmax, MTV and TLG were 4.61 ± 3.99 (0.5 ~ 17.8), 4.18 ± 6.39 (0 ~ 34.6) and 16.13 ± 28.86 (0 ~ 164.2), respectively. The PET parameters of all patients, patients with pathologic N0 and patients with pathologic N1, 2 were described in Table 3. Forty (28.8 %) patients showed MTV and TLG values of 0, because the SUVmax of the primary tumor was less than 2.0. Among those patients with MTV and TLG values of 0, no patient showed nodal metastasis.

**Table 2:** Area under the curve of MTV and TLG measured by fixed value of various SUVmax 0

Variables	Area under the curve
MTV 1.5	0.775 (0.677 ~ 0.872)
TLG 1.5	0.767 (0.672 ~ 0.863)
MTV 2.0	0.783 (0.691 ~ 0.875)
TLG 2.0	0.775 (0.685 ~ 0.866)
MTV 2.5	0.756 (0.654 ~ 0.857)
TLG 2.5	0.752 (0.650 ~ 0.853)
MTV 3.0	0.728 (0.612 ~ 0.844)
TLG 3.0	0.721 (0.605 ~ 0.836)

**Table 3:** PET parameters of 139 patients

Variables	All patients (*n* = 139)	Pathologic N0 (*n* = 115)	Pathologic N1 and N2 (*n* = 24)	*P* value^a^
SUVmax	4.61 ± 3.99	4.12 ± 3.85	6.95 ± 3.91	0.001
MTV 2.0	4.18 ± 6.39	3.09 ± 5.04	9.38 ± 9.22	0.003
TLG 2.0	16.13 ± 28.86	11.96 ± 23.72	36.10 ± 41.32	0.010

*MTV* metabolic tumor volume, *TLG* total lesion glycolysis

^a^comparison between pathologic N0 and pathologic N1, 2

**Fig. 2:**
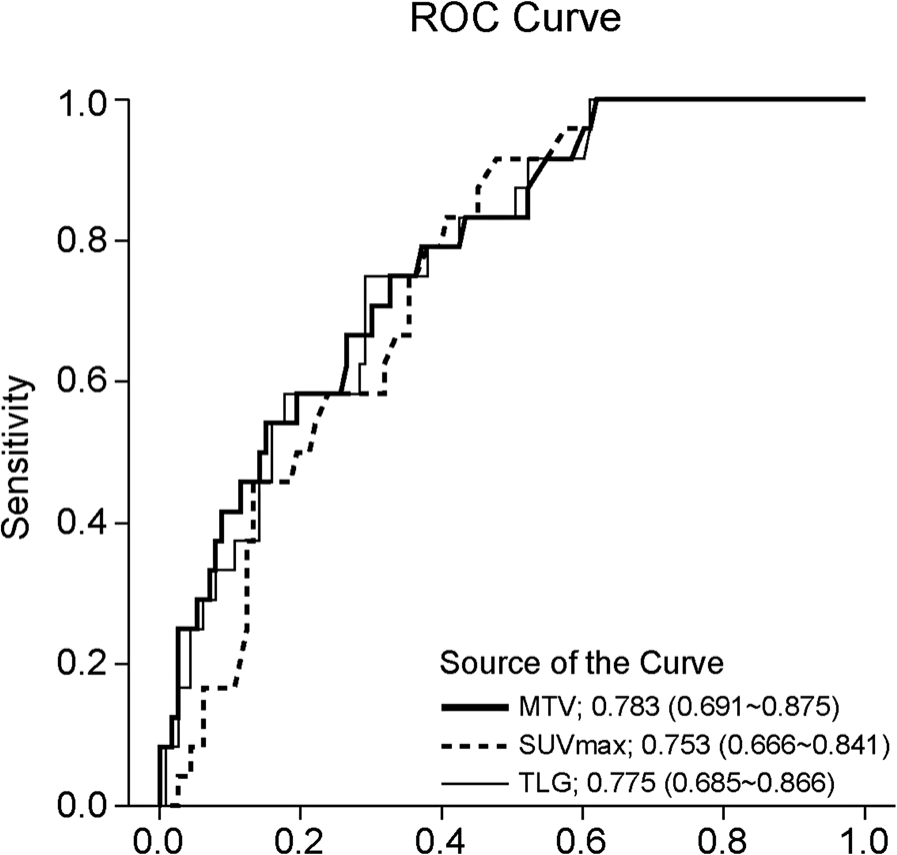
ROC curve of SUVmax, MTV and TLG for occult lymph node metastasis

In the ROC curve analysis, the AUC of MTV for occult lymph node metastasis (N1 and N2) was the highest compared with the AUCs of SUVmax and TLG (Fig. 2). The optimal cut-off values were 3.250 (sensitivity 83.3 %, specificity 60 %), 3.055 (sensitivity 75.0 %, specificity 67.8 %), and 9.829 (sensitivity 75.0 %, specificity 71.3 %) for SUVmax, MTV and TLG, respectively.

### Risk factors for occult lymph node metastasis

In univariate analysis, tumor size, SUVmax, MTV and TLG were found to be significant risk factors for occult lymph node metastasis (Table 4). Adenocarcinoma, poor differentiation, pleural dimpling and the number of dissected lymph nodes were not deemed risk factors for occult lymph node metastasis in our data.

**Table 4:** Univariate and multivairate analysis for occult lymph node metastasis

			Multivariate analysis					
	Univariate analysis		SUVmax model		MTV model		TLG model	
Variable	OR (95 % CI)	*P*	OR (95 % CI)	*P*	OR (95 % CI)	*P*	OR (95 % CI)	*P*
Tumor size (1 cm increase)	2.433 (1.089-5.436)	0.030	1.942 (0.817-4.617)	0.133	1.416 (0.570-3.518)	0.453	1.696 (0.705-4.080)	0.238
Adenocarcinoma (vs. nonadenocarcinoma)	1.611 (0.597-4.346)	0.346						
SUVmax (1 unit increase)	1.159 (1.050-1.279)	0.003	1.120 (1.003-1.250)	0.044				
MTV (1 cm^3^ increase)	1.131 (1.055-1.212)	<0.001			1.117 (1.031-1.211)	0.007		
TLG (1 unit increase)	1.022 (1.008-1.037)	0.002					1.018 (1.002-1.035)	0.031
Poor differentiation (vs. others)	2.215 (0.699-7.014)	0.176	1.473 (0.404-5.370)	0.557	0.985 (0.226 ~ 4.295)	0.984	1.202 (0.290 ~ 4.978)	0.799
Pleural dimpling (with vs. without)	1.489 (0.615-3.607)	0.378						
No. of dissected LN (1 unit increase)	1.021 (0.988-1.055)	0.223						
C-index			0.718 (0.616-0.820)		0.763 (0.660-0.856)		0.726 (0.618-0.834)	

The variance inflation factor (VIF) was calculated to investigate collinearity between tumor size and PET parameters, and was found to be 1.15 between tumor size and SUVmax, 1.17 between tumor size and MTV, and 1.15 between tumor size and TLG. Because all VIFs were < 4, collinearity between tumor size and various PET parameters was not observed.

Based on the VIFs and univariate analysis results, three different multivariate logistic regression models were established to compare the predictive power of each PET parameter. For model 1, which included tumor size, differentiation, number of dissected lymph nodes and SUVmax, the only risk factor for occult lymph node metastasis was found to be SUVmax (increase of 1 unit, odds ratio [OR] = 1.120). For model 2, which included MTV, differentiation, number of dissected lymph nodes and tumor size in the analysis, MTV (increase of 1 cm^3^, OR = 1.117) was also found to be the only risk factor for occult lymph node metastasis. For model 3, which included TLG, differentiation, the number of dissected lymph nodes and tumor size, TLG (increase of 1 unit, OR = 1.018) was not found to be a significant risk factor for occult lymph node metastasis (Table 4).

The C-index was calculated for each multivariate logistic regression model. The C-indices of models 1, 2 and 3 were 0.718 (95 % CI 0.616 – 0.862), 0.763 (95 % CI 0.660 – 0.856), and 0.726 (95 % CI 0.618 – 0.834), respectively (Table 4). The C-indices of the MTV model (model 2) showed the highest C-index, even though statistical significances were not observed.

## Discussion

This present study demonstrated that the MTV of a primary tumor is an important predictor of lymph node metastasis in small-sized and clinically node-negative NSCLC. MTV showed a better predictive performance than that of SUVmax or TLG, although the differences were not found to be statistically significant. This study may be the first investigation of the predictive value of volume-based parameters on lymph node metastasis in small-sized and peripheral NSCLC. Our results suggest that MTV helps indicate adequate surgical extent and identify possible candidates for sublobar resection.

Anatomic resection, such as lobectomy and mediastinal lymph node dissection, has been regarded as the default surgery for several decades [17], and sublobar resection, such as wedge resection and segmentectomy, has been performed selectively in patients with old, with poor lung function and early-stage NSCLC [7]. The question has recently arisen as to whether sublobar resection is an oncologically valid procedure. Several retrospective studies and recent meta-analyses have reported that anatomic segmentectomy could offer similar outcomes compared with lobectomy, in terms of both overall and disease-free survival [18]. Even though there has been no exact consensus on the indications for sublobar resection, several criteria have been suggested: confirmed stage IA disease only, small tumors up to 2–3 cm in diameter and a peripheral tumor location within the lung [17]. In addition, the presence of nodal metastasis is also important; if nodal metastasis is present, lobectomy and mediastinal lymph node dissection, instead of sublobar resection, is mandatory. Therefore some thoracic surgeons insist that intraoperative frozen sections should be examined for all hilar and lobe-specific mediastinal lymph nodes to confirm the intraoperative N staging as N0 during sublobar resection [1, 4, 6]. However, intraoperative examination of many lymph nodes is unrealistic and difficult for thoracic surgeons and pathologists in the clinical setting. If pN0 can be predicted from preoperative information, sublobar resection without strict intraoperative lymph node assessment can be performed in patients with early NSCLC [8]. Tsutani et al. reported solid tumor size and SUVmax as significant independent predictors of nodal involvement in clinical stage IA lung adenocarcinoma; a solid tumor size < 0.8 cm on HRCT or an SUVmax < 1.5 on FDG-PET/CT may be helpful for avoiding systematic lymphadenectomy [8]. However, the AUC for SUVmax on ROC curve analysis in a previous study was only 0.761 (95 % CI 0.703-0.819). Therefore, accurate parameters other than SUVmax and solid tumor size are needed.

The clinical significance of volume-based PET parameters has been widely investigated in NSCLC [9–14, 19, 20]. MTV indicates the volume of a metabolically active tumor, and TLG is the product of the mean SUV and MTV; thus, these parameters reflect tumor burden. These parameters can be measured rapidly and consistently in the clinical field with the advance of image analysis software. SUVmax is also a useful parameter and may be a surrogate marker for tumor aggressiveness [21, 22]; however, it is affected by many factors and is highly sensitive to noise [23–25]. Recent studies have demonstrated the prognostic value of volume-based parameters in early stage NSCLC [12–14]; therefore, tumor volume may be important not only in advanced-stage but also early-stage NSCLC. Regarding the prediction of lymph node metastasis, two recent studies have been reported. Lebioda et al. reported that izoSUV2.5 volume (the volume of primary tumor inside SUV 2.5 line) was a risk factor for mediastinal lymph node involvement after analyzing the 70 NSCLC patients [26]. However, they analyzed heterogenous group of patients with cT1-4 N0-1 lesions. Kim et al. reported that metabolic parameters including volume-based parameters were significant predictors of occult lymph node metastasis in clinically node-negative squamous cell lung carcinoma; this study analyzed large-sized tumors (cT1-3 N0 NSCLC) in 63 patients [19]. However, the clinical implication of occult lymph node metastasis is significant in cT1 NSCLC, because its absence can serve as an indication for sublobar resection. Anatomic resection, such as lobectomy and complete mediastinal lymph node dissection, is the treatment of choice in cT2-3 N1-2 NSCLC. Therefore, we focused on small peripheral NSCLC < 3 cm. As we hypothesized, MTV showed a slightly higher AUC in the ROC curve analysis and C-index in the multivariate analysis, which indicated a higher predictive value than that of SUVmax in small peripheral NSCLC, even though the statistical differences were not significant in our data. In our study, TLG showed a lower AUC than those of MTV and SUVmax, and these results are consistent with a previous study reporting that TLG is not a risk factor for nodal metastasis in multivariate analysis [19].

Even though MTV showed promising results as a predictor of occult lymph node metastasis in small NSCLC, this study has to be interpreted with caution. SUVmax remains a valuable predictor for detecting lymph node metastasis and was verified in a previous study involving a large number of patients [8]. Even though MTV showed a slightly higher C-index than that of SUVmax, volume-based parameters such as MTV have an important limitation. Due to the calculation method of volume-based parameters, patients with low SUVmax could be underestimated, based on the cut-off value. Even though 40 patients with zero MTV and TLG showed no occult lymph node metastasis in this study, the possibility of underestimation by volmue-based parameters has to be kept in mind.

This study has several limitations. First, it was a retrospective, single-center study and the study population was relatively small. Although the AUC and C-index of MTV were found to be slightly higher those of SUVmax, they were not statistically significant. These results might originate from the small number of enrolled patients, and thus further analysis using a sufficient number of patients is required for exact comparisons of the AUC and C-index. Second, the type of operation was not uniform although patients predominantly underwent lobectomy with mediastinal lymph node dissection. However, because all patients underwent meticulous mediastinal lymph node dissection, undiagnosed lymph node metastasis might not be present. Third, pleural invasion was not a risk factor for occult lymph node metastasis although several previous studies have reported that pleural invasion itself is a risk factor for survival after surgery [27]. Whether pleural invasion is related to occult lymph node metastasis needs to be evaluated in further analyses using sufficient numbers of patients. Forth, we did not perform partial volume correction in our study dealing with small-sized tumor. However, the patients who had the tumor less than 1.0 cm were 12 (8.6 %) in the present study, which may not cause significant effects on our results. Finally, thin-section CT findings except tumor diameter were not considered in this study. Despite these limitations, this study is the first to analyze volume-based parameters as risk factors for occult lymph node metastasis in small and early NSCLC. In addition, the advantage of this study lies in the use of the C-index of several multivariate logistic regression models to compare the importance of PET parameters as predictors of occult lymph node metastasis.

## Conclusion

The SUVmax and volume-based parameters of primary lesions were found to be significant risk factors for nodal metastasis in small peripheral NSCLC. MTV showed a better predictive performance than did the other PET parameters; therefore, MTV may be a possible indicator for sublobar resection in clinically node-negative small-sized NSCLC. A further study using a larger cohort of patients is needed to validate the predictive role of these biomarkers derived from FDG PET.
